# Outbreak of Acute Fulminant Myocarditis in Children in Campania Region, Italy: A Case Series

**DOI:** 10.3390/children11121414

**Published:** 2024-11-23

**Authors:** Antonietta Giannattasio, Marco Maglione, Giangiacomo Di Nardo, Giovanni Maria Di Marco, Daria Lauretta, Maria Chiara Carrella, Daniela Furlan, Fabio Savoia, Vincenzo Tipo

**Affiliations:** 1 Pediatric Emergency Unit, Santobono-Pausilipon Children’s Hospital, 80129 Naples, Italy; m.maglione@santobonopausilipon.it (M.M.); mariachiara.carrella@unina.it (M.C.C.); d.furlan@santobonopausilipon.it (D.F.); enzotipo@libero.it (V.T.); 2 Pediatric Cardiology Unit, Santobono-Pausilipon Children’s Hospital, 80129 Naples, Italy; g.dinardo@santobonopausilipon.it (G.D.N.); gm.dimarco@santobonopausilipon.it (G.M.D.M.); d.lauretta@santobonopausilipon.it (D.L.); 3 Childhood Cancer Registry of Campania, Santobono-Pausilipon Children’s Hospital, 80129 Naples, Italy; f.savoia@santobonopausilipon.it

**Keywords:** fulminant myocarditis, children, outbreak

## Abstract

Acute fulminant myocarditis is a rare event in children, accounting for about 10% of all cases of acute myocarditis. Its lack of specific onset patterns and unpredictable evolution make diagnosis and prompt treatment challenging. We observed six cases of fulminant myocarditis admitted to our Pediatric Emergency Unit (Campania region, Sothern Italy) within a very short timeframe (50 days, from July to September 2024). Three of them died, and two are still under treatment in a Pediatric Cardiologic Intensive Care Unit in critical condition. In only one case, cardiac function improved. The described cases were not geographically linked, belonging to different areas of Southern Italy. No common etiological agent was found. Given the relatively low incidence of the condition, the occurrence of six pediatric myocarditis within approximately two months should be considered exceptional. Careful monitoring of further cases in the next few months should be warranted.

## 1. Introduction

Acute myocarditis is a rare event in children, accounting for 0.05% of hospitalized patients [1]. It may present with symptoms ranging from a mild clinical picture, with gastrointestinal or upper respiratory tract involvement and chest pain, to life-threatening arrhythmias up to severe cardiogenic shock and sudden death [2]. The lack of a specific onset pattern and the unpredictable evolution make diagnosis and prompt treatment of myocarditis challenging. Age distribution displays a bimodal pattern with peaks at 1 year and during adolescence, with a more severe clinical course in young children [3]. The incidence of myocarditis is not related to seasonality, and it is approximately the same both in the winter months (the season with a high rate of viral infections) and in spring or summer [4].

Acute myocarditis commonly has an infectious cause, with viruses being the most prevalent triggers [5]. However, it may also accompany autoimmune diseases or other conditions [5,6]. Among infections, enteroviruses have classically represented the main viral agent, but also other viruses, including Adenovirus, Parvovirus B19, Human Herpesvirus 6, Epstein–Barr virus (EBV), and Cytomegalovirus, have been recently identified by means of polymerase chain reaction (PCR) in myocardial tissues from patients with myocarditis [5]. Mechanisms of myocardial damage may significantly vary according to the involved pathogen. For instance, after viral entry into the myocytes through a specific cell-surface receptor, enteroviruses activate innate immunity, start replication, and induce an acquired immune response leading to myocardial dysfunction [7]. Similarly, a common transmembrane receptor allows Coxsackie B viruses and Adenoviruses entry into the myocytes, but antigens deriving from cellular degradation trigger immune response and cytokine production, which potentiate cardiac damage [7].

Fulminant myocarditis is a rare, aggressive myocarditis characterized by rapid clinical deterioration with severe heart failure, refractory arrhythmias, and cardiogenic shock, often leading to death [8]. It accounts for about 10% of adult cases of acute myocarditis [9] and for 30–40% of cases of myocarditis occurring in childhood [10]. Treatment includes pharmacological and/or mechanical circulatory support (MCS) [5]. Despite possible fatality in its acute phase, when aggressive hemodynamic support is required, its long-term prognosis seems to be good in adults [9]. This likely happens because fulminant presentation is a marker of a more robust immunological/inflammatory response that is associated with a more effective viral clearance and is thus predictive of complete myocardial recovery once the acute phase is resolved [11].

Fulminant myocarditis should be identified at its earliest stages using the most relevant advanced diagnostic modalities to provide prompt treatment. Its management includes emergency stabilization of clinical conditions and even transfer of these patients to centers equipped with facilities able to guarantee full circulatory support.

Herein, we describe six children who experienced fulminant myocarditis over a very short timeframe.

## 2. Methods

### Diagnosis of Fulminant Myocarditis

Fulminant myocarditis was defined as a myocarditis characterized by sudden and significant severity, resulting in an exceptionally high risk of death caused by cardiogenic shock, fatal ventricular tachyarrhythmias or bradyarrhythmias, requiring vasoactive drug support or temporary MCS [11]. The diagnosis in our cases was not based on endomyocardial biopsy (EMB) results because of the hemodynamic instability and rapidly progressive worsening of clinical conditions. Therefore, the diagnosis was based on coherent clinical, biochemical, and instrumental data and supported by the exclusion of alternative causes of cardiogenic shock.

To assess the etiology of fulminant myocarditis, each patient underwent a nasal swab that was analyzed by means of PCR (FilmArray^®^ Respiratory Panel BioFire Diagnostics LLC 390; Wakara Way, Salt Lake City, UT, USA) for a wide panel of respiratory viruses: Respiratory Syncytial Virus, Human Parainfluenza Viruses 1–4, Adenovirus, Rhino/enteroviruses, Metapneumovirus, Human Coronaviruses, Middle East Respiratory Syndrome Virus, Severe Acute Respiratory Syndrome Coronavirus-2 (SARS-CoV-2), Bordetella pertussis and parapertussis, Chlamydia pneumoniae and Mycoplasma pneumoniae. Furthermore, whenever possible, serology for Mycoplasma pneumoniae, EBV, TORCH, and Parvovirus B19 was performed. In selected cases, a targeted DNA search for EBV, Adenovirus, and Parvovirus B19 on blood samples was added.

## 3. Results

### 3.1. Outbreak of Fulminant Myocarditis 

In the period between 28 July and 19 September 2024, 6 children with fulminant myocarditis were admitted to the Pediatric Emergency Unit of Santobono-Pausilipon Children’s Hospital (Naples, Campania Region, Southern Italy). This is the only pediatric hospital equipped with a Pediatric Emergency Department in the Campania region and serves a large, varied pediatric population (aged < 14 years). This hospital is also equipped with a Pediatric Cardiology Unit and a Pediatric Intensive Care Unit (PICU) but cannot provide extracorporeal membrane oxygenation (ECMO) whenever necessary.

To compare the number of children diagnosed with myocarditis at our center in the first 8 months of 2024 versus 2023, we screened the hospital medical record system databases using the discharge diagnostic code (“myocarditis”) from the International Classification of Diseases—Tenth Revision. We found 18 cases of acute myocarditis diagnosed during the period January–August 2024 (including 5 out of 6 patients described below) and only 6 cases within the same period of 2023. The number of cases of myocarditis observed in the first 8 months of 2024 was higher than the number of cases per year admitted since 2016 (3 cases in 2022, 5 in 2021, no patient in 2020, 9 cases in 2019, 4 in 2018, 6 in 2017, 7 in 2016 and 11 cases in 2015).

### 3.2. Case Series

All patients but one were otherwise healthy and developed severe hemodynamic impairment requiring high doses of vasopressors or ECMO. Patients’ characteristics are reported in Table 1. The 6 cases were not geographically linked and belonged to different areas of Southern Italy (5 children came from the Campania region, one from Isernia, Molise region). No patient underwent cardiac magnetic resonance (CMR) or EMB.

Patient 1 was a 4-month-old girl admitted to our Emergency Department because of fever, diarrhea, and poor appetite. At admission, the infant was assisted in the shock room because of the rapid worsening of general conditions. Echocardiography demonstrated severe cardiac dysfunction with left ventricular dilatation, mitral and tricuspid valve regurgitation, and absence of pericardial effusion. She received one dose of ceftriaxone, one dose of dexamethasone (0.2 mg/kg), dobutamine, and three cycles of advanced cardiopulmonary resuscitation. The child experienced cardiogenic shock and died after 2 h. SARS-CoV-2 was detected on a nasopharyngeal swab. Given the extremely rapid evolution, other etiologic investigations were not performed on this child.

Patient 2, a 4-year-old boy, was referred to our Pediatric Emergency Department from a local hospital because of severe dyspnea. One week before hospital admission, he had presented a fever for 3 days, followed by vomiting and abdominal pain. At admission, point-of-care echocardiography revealed a diffuse reduction in motion amplitude in the left ventricular wall, mild left ventricular dilatation, and functional mitral and tricuspid valve regurgitation. Prompt treatment with dobutamine (2 mcg/kg/min) and methylprednisolone (1 mg/kg) was started. After stabilization in the shock room, the child was transferred to the regional reference hospital with facilities providing ECMO. Here, treatment with adrenalin (0.025 mcg/kg/min) and enoximone (10 mcg/kg/min) was added. He also received intravenous immunoglobulins (IVIg), antibiotics, and diuretics. His cardiac function progressively improved, and he was discharged after 38 days of hospitalization. Etiological laboratory investigation showed positive anti-Parvovirus B19 IgM and IgG and positive blood PCR for Parvovirus B19 DNA.

Patient 3 was a 12-month-old girl born in Guinea, admitted to our Emergency Department because of a suspected seizure. At admission, electrocardiography (ECG) showed supraventricular tachycardia. Echocardiography demonstrated a severe reduction in global ventricular systolic function with left ventricular dilatation and moderate mitral and tricuspid valve regurgitation. She received two boli of adenosine (0.2 mg/kg) without resolution of the arrhythmia. Cardioversion was obtained with amiodarone (5 mg/kg) administration. After endotracheal intubation, the child was admitted to our PICU. She experienced a rapid disease progression with cardiogenic shock and lethal arrhythmia and died after 5 h. An autopsy is ongoing on this child.

**Figure 1:**
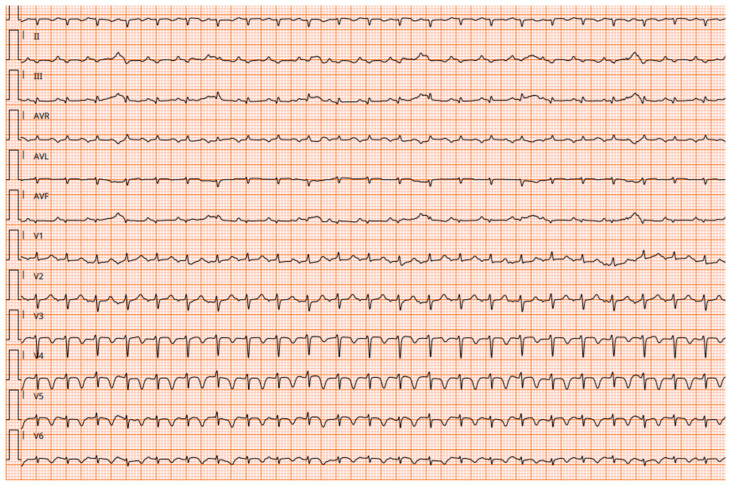
ECG at admission from patient 4. Low-voltage complexes and diffuse ventricular repolarization abnormalities.

Patient 4 was a 17-month-old boy admitted to a local hospital because of fever and vomiting. He was promptly transferred to our Emergency Department because of his worsening clinical condition. At admission, ECG showed low-voltage complexes and diffuse ventricular repolarization abnormalities (Figure 1). 

**Figure 2:**
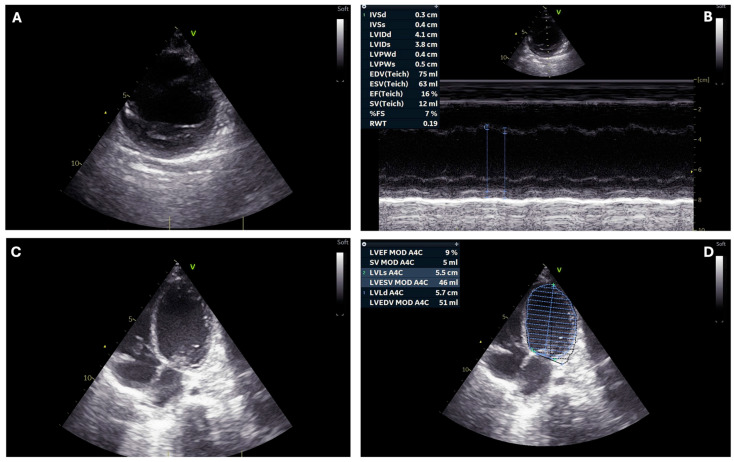
Echocardiography from patient 4. Parasternal short axis (panel **A**) and four-chamber (panel **C**) views show severe dilatation of the left ventricle. Ventricular hypokinesia was assessed by means of M-Mode (panel **B**) and Biplane Simpson Method (panel **D**).

Point-of-care echocardiography exhibited globally compromised ventricular systolic function with a ventricular ejection fraction of 10% (Figure 2). 

Dobutamine (1 mcg/kg/min) was started. The child was intubated and transferred to our PICU, where he received intravenous steroids, inotropes, and IVIg. Given the lack of improvement and persistent reduction in ventricular ejection fraction (10%), he was referred to the regional reference hospital for ECMO. Despite MCS, he died after 19 days. Etiologic investigations showed a positive nasopharyngeal swab for Mycoplasma pneumoniae. All other etiologic investigations resulted in negative outcomes.

Patient 5 was a 2-year-old male admitted to our Pediatric Emergency Department because of dyspnea. Chest X-ray showed cardiomegaly and cardiac evaluation was therefore performed in the shock room. The echocardiogram showed a severe reduction in ventricular function with left ventricular and atrial dilatation and moderate functional aortic and mitral valve regurgitation. After admission to our PICU, inotropes (dobutamine 8 mcg/kg/min) and diuretics were started. He also received IVIg, dexamethasone, antibiotics, and diuretics. Because of the lack of improvement in clinical and echocardiographic findings, he was transferred to the regional reference hospital with an ECMO facility. In the following weeks, his cardiac function slightly improved. He did not undergo MCS, but he is still hospitalized. As for etiologic investigations, Adenovirus and Parainfluenza 4 virus were identified on nasopharyngeal swabs. Serology and blood PCR were negative for other pathogens.

Patient 6 was addressed to our Pediatric Emergency Department because of vomiting, dehydration, and poor general conditions. At admission, an echocardiogram showed a severe reduction in ventricular function dilatation of the left ventricle, moderate mitral valve regurgitation, and minimal pleural effusion. Based on the severely impaired cardiac function, he was promptly transferred to the regional reference hospital with an ECMO facility. Here, he received inotropes, IVIg, and one bolus of a high dose of methylprednisolone (30 mg/kg/day), with a very slight improvement in ventricular ejection fraction to date (20%). Serologic and molecular tests for putative etiologic agents were all negative. Urine culture resulted positive for Escherichia coli spp.

## 4. Discussion

Fulminant myocarditis is a life-threatening event that may present as cardiogenic shock. Tachyarrhythmias are common, and supportive measures with mechanical ventilation, inotropic agents, and vasopressors to correct hypotension, respiratory failure, and overt cardiogenic shock are often needed. Some patients require MCS [5,12,13,14]. The present study moved from the observation of an unusual concentration of cases of fulminant myocarditis in a single center within a limited timeframe. We described the etiology, clinical course, and laboratory characteristics of these patients and aimed to raise physicians’ awareness of this condition to warrant timely recognition and treatment.

### 4.1. Etiology

The role of the identified etiologies of fulminant myocarditis deserves consideration, as different pathogens act differently in determining cardiac damage, and this may affect clinical presentation and outcomes. In our cohort, given the very rapidly progressive evolution observed in all patients, specific correlations between infectious agents and the clinical picture are tough to establish, and the concentration of a series of myocarditis with such heterogeneous etiologies in one center remains unexplained. Nevertheless, it is noteworthy that similar clinical pictures derive from pathogens whose mechanisms of action are deeply different. 

Parvovirus B19 has been reported to cause fulminant myocarditis both in children and adults [15,16]. Interestingly, the primary viral target has been shown not to be the cardiomyocyte but the endothelial cells, whose infection is followed by cytokine production and induction of apoptosis, ultimately leading to endothelial dysfunction. Myocardial is thus secondarily involved, and its damage likely derives from T lymphocyte recruitment with subsequent inflammation, local hypoperfusion, or ischemia [17].

With regards to SARS-CoV-2, several reports have supported the association between this pathogen and myocardial impairment, including fulminant forms in young patients [18,19]. It is not clear whether SARS-CoV-2-related myocardial damage directly derives from its intracellular activity after binding to the angiotensin-converting enzyme 2 receptors expressed on myocytes or rather from a hyperimmune response. It is likely that the two mechanisms act synergically, with the first probably leading to myocardial impairment during acute infection and the second dominating the pathogenesis of SARS-CoV-2-related Multisystem Inflammatory Syndrome [20].

Unlike the previously mentioned viral pathogens, Mycoplasma pneumoniae represents a less common cause of acute myocarditis in children, with cardiac dysfunction occurring in less than 5% of infected patients [21]. Evidence regarding the pathogenesis of myocardial damage in Mycoplasma pneumoniae infections is scant. Nevertheless, some authors have proposed the role of autoimmune modulations with the production of antiphospholipid antibodies as a possible mechanism leading to thrombosis and, ultimately, to myocardial dysfunction [22,23].

As for Adenovirus-related myocarditis, the infection has been reported to determine a reduction in gap junction intercellular communication, thus affecting conduction and creating an arrhythmogenic substrate. However, likewise, with other pathogens, immune-mediated effects cannot be ruled out [24].

### 4.2. Diagnosis

With regards to clinical presentation, in our case series, fever and vomiting were the most common presenting symptoms, in line with previously published findings [25]. As acute gastroenteritis presenting with fever, vomiting, and diarrhea is extremely common in children, diagnosis of acute myocarditis still represents a challenge for physicians.

The gold standard for the diagnosis of myocarditis is the percutaneous EMB, which allows the demonstration of inflammatory infiltrates in myocardial tissue [11,26]. Also, in fulminant myocarditis, EMB has been identified as an accurate diagnostic tool as it may allow the detection of diffuse myocardial inflammation. Furthermore, it is of relevance when specific conditions like eosinophilic myocarditis or cardiac sarcoidosis are suspected. Nevertheless, these forms of myocarditis, which may present with a fulminant onset and require a targeted therapeutic approach, are not common in children [26]. 

EMB is not considered mandatory, especially in cases of pediatric myocarditis [27]. For this reason, and due to its invasiveness, we did not require EMB for a definite diagnosis of myocarditis in our patients. Rather, we took into account clinical signs, chest X-ray, ECG, echocardiography, and laboratory investigations. No biopsy was performed, even in the patient who underwent ECMO. Furthermore, and unfortunately, myocardial preparation from the child who underwent autopsy was not available. Therefore, we could not discuss correlations between etiology and type of tissue damage, and this represents undoubtedly a major limitation of the present study.

More recently, contrast-enhanced CMR has provided unique insights into tissue-level pathology consistent with myocarditis, including myocardial edema and fibrosis [11]. Nevertheless, CMR may not be considered at an early stage in the diagnostic algorithm of fulminant myocarditis, as it is poorly feasible in the acute phase of patient instability (>10 days later than in non-fulminant myocarditis). Such a technique is primarily useful in the characterization of myocardial impairment once the diagnosis has been made and the patient has been stabilized.

An abnormal ECG is reported in about 70% of cases of acute myocarditis [12], although there are no ECG findings specific to myocarditis, and the sensitivity of ECG is variable (47–85%) [10]. In patients with myocarditis, ECG may show a wide range of findings, such as tachycardia, low voltage, QTc prolongation, flat/negative T wave, and ST-T abnormalities. When inflammation affects the impulse-conducting system, arrhythmia reflecting ventricular conduction disturbance, atrioventricular block, ventricular tachycardia, and fibrillation may appear [10].

As for laboratory investigations, troponin, B-type natriuretic peptide (BNP), and inflammatory markers (mainly C-reactive protein, CRP) should be performed in case of suspicion of acute myocarditis. Although troponin elevation is not correlated with the severity of cardiac dysfunction or arrhythmias, higher troponin levels have been associated with higher mortality [5]. In our cases, troponin levels were not related to the outcome; however, the only patient with the quickest improvement of ventricular ejection fraction (Patient 2) had the lowest troponin value. BNP was elevated at the time of presentation in all children in which it was available (5/6 cases). A high BNP is associated with cardiac dysfunction, signs of acute heart failure, and the need for cardiopulmonary resuscitation [5]. However, increased BNP levels are related to heart failure and not specifically to myocarditis. CRP was normal (or slightly increased) in all patients at presentation. This is in line with previous reports, underlying the concept that normal levels of inflammatory markers do not exclude myocarditis [28]. 

Routine viral serologies should be performed in all cases of acute myocarditis. However, they have a low sensitivity and specificity. The search for viral genome by PCR, particularly when performed on EMB samples, is far more accurate [28]. However, this investigation is limited by the invasiveness of the procedure. Furthermore, viruses in EMB are detected in about half of cases [29]. Recently, Zhao et al. retrospectively described the clinical characteristics and outcomes of 79 children with acute fulminant myocarditis observed over a ten-year period [25]. In this series, a putative etiology was found in about 75% of cases. Viruses were the most common pathogens, followed by Mycoplasma pneumoniae [25]. We found an etiology in four (66.6%) cases (SARS-CoV-2, Parvovirus B19, Mycoplasma pneumoniae, and Adenovirus). In the two patients who died a few hours after admission (Patient 1 and Patient 3), no extensive etiologic investigation was performed due to the rapidly fatal evolution. Unfortunately, the results of the autopsy performed on patient number 3 are still not available.

Unlike adults, differential diagnosis of acute myocarditis in children is easier, given the limited role of confounding factors such as cardiac co-morbidities and coronary artery disease [26]. Alternative diagnoses to be considered in patients with suspected fulminant myocarditis mainly include sepsis, hypovolemic shock, and cardiac structural abnormalities. Discriminating sepsis from early cardiogenic shock secondary to myocarditis is challenging during the early stages of workup and treatment, also because patients with myocarditis are often febrile. Early recognition of circulatory compromise, such as narrow arterial pulse pressure, sinus tachycardia, and cool or mottled extremities, is mandatory. Rapid worsening of clinical conditions, respiratory distress, lethargy, and vomiting without diarrhea should be considered as signs of high suspicion for myocarditis. Echocardiography remains the first test in most cases of fulminant myocarditis, as it allows one to rapidly rule out a wide spectrum of differential diagnoses, including pericardial disease, and to assess both cardiac morphology and function. In our cases, point-of-care echocardiography performed in the shock room of the Emergency Department allowed us to promptly diagnose myocarditis and establish the severity of cardiovascular compromise in order to start appropriate treatment.

### 4.3. Treatment

In the absence of well-established treatment guidelines specifically tailored to children, current strategies are symptom-based therapies largely aimed at managing heart failure and arrhythmias. Strict echocardiographic monitoring is necessary to ensure timely treatment because the condition may rapidly progress within a few hours. Patients with low cardiac output not responding to maximal pharmacological therapy should be immediately referred to facilities able to provide MCS [10]. In the presence of early signs of circulatory failure, a direct transfer from the Emergency Department to a tertiary care facility with expertise in advanced circulatory support and transplantation should be considered. Starting MCS before cardiovascular collapse is expected to increase the likelihood of survival and a better prognosis with a return to normal cardiac function [10]. In our series, all patients promptly received inotropes. Two patients (patients 1 and 3) died a few hours after admission to the Emergency Department. The other two children (patients 4 and 5) were transferred to the reference center for MCS after a few days of hospitalization in the PICU due to a lack of improvement in cardiac function. The remaining two cases were stabilized in the shock room of the Emergency Department and then transferred for eventual MCS.

Although specific treatment of fulminant myocarditis encompasses antiviral agents, steroids, and IVIg, a specific guideline regarding the indications and doses of these agents in children is not available [29]. IVIg is an important immunomodulatory treatment option, and some studies suggest that its use is related to better outcomes in patients with fulminant myocarditis [30,31]. In our series, IVIg was used in 4/6 patients. In the remaining two children (patients 1 and 3), showing a rapidly deteriorating clinical course and death in a few hours, no therapy other than cardiocirculatory support was administered.

Immunosuppression therapy, often involving steroids alone or in combination with medications like azathioprine or cyclosporine, has proven useful in improving left ventricular ejection fraction in cases of virus-negative myocarditis [32]. Immunosuppressors are the mainstay of treatment for specific conditions like eosinophilic myocarditis and fulminant myocarditis associated with systemic autoimmune diseases [8]. Nevertheless, their effectiveness in children with viral myocarditis has not been confirmed [33]. In these cases, their use is limited by the possibility of increased viral replication and worsening of myocardial damage. Steroids were used in 5/6 of the described patients. In 3 cases (patients 4, 5, and 6), this therapy was started a few days after admission, whereas in the other two children (patients 1 and 2), steroids were started earlier because of worsening clinical conditions. The only patient who did not receive steroids was the child (patient 3) with a rapidly fatal outcome. No other immunosuppressive agents were used in our series.

When a viral cause is confirmed, targeted therapies may offer effective treatment options. Interferon β therapy has shown promising results in cases of Adenovirus or enterovirus-induced myocarditis [32], but it was not administered in any of the reported patients.

## 5. Conclusions

Fulminant myocarditis is a medical emergency that can have an extremely rapid progression and a poor prognosis, with the need for prompt inotropic support or even MCS. Given the relatively low incidence of the condition, the occurrence of six cases of pediatric fulminant myocarditis in less than two months should be considered exceptional. Even though the casual concentration of cases in a short timeframe may not be excluded, careful monitoring of further cases is mandatory in the next months to identify a potentially emerging serious infectious threat. To date, no regional or national tracking system for myocarditis is available in our country. Nevertheless, moving from our experience, such a condition has entered the national pediatric surveillance system established within the network of a European Union-funded National Recovery and Resilience Plan project, the One Health Basic and Translational Actions Addressing Unmet Needs on Emerging Infectious Diseases (INF-ACT) [34]. As prompt recognition of the condition is crucial to start appropriate and possibly life-saving treatment, physicians operating both in hospital and outpatients’ settings should keep in mind this diagnosis to correctly face the condition as soon as possible to avoid the progression towards cardiogenic shock and irreversible cardiac function impairment. For institutions not equipped with facilities for advanced heart failure surgical and medical management, it is important to refer patients with suspected fulminant myocarditis directly from the Emergency Department to a tertiary care referral center. 

## Figures and Tables

**Table 1:** Clinical, laboratory, and cardiac findings at admission in six patients with fulminant myocarditis.

	Patient 1	Patient 2	Patient 3	Patient 4	Patient 5	Patient 6
Age	4 months	4.6 years	1 year	1.5 years	2 years	1.4 years
Gender	F	M	F	M	M	M
Chronic conditions	Moderate interatrial defect	No	No	No	No	Ureterectasia and pyelectasis
Symptoms before hospitalization	Fever, diarrhea	Fever, vomiting, abdominal pain	Vomiting	Fever, vomiting	Dyspnea	Vomiting
Duration of symptoms before hospitalization	Few hours	One week	Few hours	Three days	One week	Few hours
C-reactive protein (mg/L; normal value < 5)	Negative	Negative	Negative	11.2	Negative	26.97
Procalcitonin	Negative	Negative	Negative	Negative	Negative	Negative
Aspartate aminotransferase (IU/L)	73	Not available	Non-available	424	30	611
Lactate dehydrogenase (U/L)	416	Not available	Not available	1042	358	1344
Lactate (mmol/L)	14.9	3.6	13.9	16	4.2	5.1
Troponin (ng/L)	202	51	573	130	115	468
B-type natriuretic peptide (pg/mL)	Not available	2820	3730	4340	3270	>5000
CK-MB (ng/mL; normal value < 6.2)	2.7	7.36	Not available	25.64	4.2	Not available
Myoglobin (ng/mL; normal value 20–72)	147	Not available	Not available	4167	34.08	Not available
Chest X-ray	Reticular-nodular pattern, cardiomegaly	Reticular-nodular pattern, bilateral pleural effusion	Reticular-nodular pattern	Not performed	Cardiomegaly	Not performed
Blood pressure (mmHg)	Undetectable	87/60	Undetectable		110/60	93/69
Electrocardiography	Sinus tachycardia	Atrioventricular conduction delays	Supraventricular tachycardia and fatal ventricular tachycardia	Atrioventricular conduction delays	Sinus tachycardia, low voltage QRS complex	Non-specific ventricular repolarization abnormalities
Ventricular ejection fraction (%)	<10	25	10–15	10–15	33–35	10–15
Outcome	Death	Discharged	Death	Death	Still hospitalized (Cardiologic Intensive Care Unit)	Still hospitalized (Cardiologic Intensive Care Unit)

## Data Availability

The data presented in this study are available on request from the corresponding author. The data are not publicly available due to privacy restriction.
